# Adaptation to Emotional Conflict: Evidence from a Novel Face Emotion Paradigm

**DOI:** 10.1371/journal.pone.0075776

**Published:** 2013-09-20

**Authors:** Peter E. Clayson, Michael J. Larson

**Affiliations:** 1 Department of Psychology, University of California Los Angeles, Los Angeles, California, United States of America; 2 Department of Psychology, Brigham Young University, Provo, Utah, United States of America; 3 Neuroscience Center, Brigham Young University, Provo, Utah, United States of America; University of Jyväskylä, Finland

## Abstract

The preponderance of research on trial-by-trial recruitment of affective control (e.g., conflict adaptation) relies on stimuli wherein lexical word information conflicts with facial affective stimulus properties (e.g., the face-Stroop paradigm where an emotional word is overlaid on a facial expression). Several studies, however, indicate different neural time course and properties for processing of affective lexical stimuli versus affective facial stimuli. The current investigation used a novel task to examine control processes implemented following conflicting emotional stimuli with conflict-inducing affective face stimuli in the absence of affective words. Forty-one individuals completed a task wherein the affective-valence of the eyes and mouth were either congruent (happy eyes, happy mouth) or incongruent (happy eyes, angry mouth) while high-density event-related potentials (ERPs) were recorded. There was a significant congruency effect and significant conflict adaptation effects for error rates. Although response times (RTs) showed a significant congruency effect, the effect of previous-trial congruency on current-trial RTs was only present for current congruent trials. Temporospatial principal components analysis showed a P3-like ERP source localized using FieldTrip software to the medial cingulate gyrus that was smaller on incongruent than congruent trials and was significantly influenced by the recruitment of control processes following previous-trial emotional conflict (i.e., there was significant conflict adaptation in the ERPs). Results show that a face-only paradigm may be sufficient to elicit emotional conflict and suggest a system for rapidly detecting conflicting emotional stimuli and subsequently adjusting control resources, similar to cognitive conflict detection processes, when using conflicting facial expressions without words.

## Introduction

Cognitive control is necessary to focus attention on pertinent information in order to strategically adapt to changes in the environment [1]. Effective regulation of emotions similarly requires the ability to appropriately detect emotional content and adjust action in order to approach or avoid appetitive or aversive stimuli [2,3]. A prevailing theoretical explanation of the recruitment of top-down control of cognitive and emotional information highlights the role of conflict processing in the regulation of control processes [4-7]. Conflict processing refers to the ability to detect and signal when competing stimulus- or response-options are present. Support for the role of conflict processing in both cognitive and emotional contexts is grounded in findings from tasks where task-relevant and task-irrelevant information queue either identical responses (i.e., congruent trial, low conflict) or opposing responses (i.e., incongruent trial, high conflict) [8]. Emotionally salient conflicting information may signal the recruitment of control similarly to more traditional tasks that involve cognitive conflict (such as the incongruent condition of the Stroop task); however, the preponderance of research examining emotional conflict to date utilizes a combination of emotional word (i.e., lexical stimuli) and face stimuli to investigate this possibility [6,9].

Cognitive and affective information interact and recruit attention from a similar pool of resources to implement top-down control [10]. As such, our understanding of the mechanisms required to recruit emotional control in response to emotional conflict may be distorted and misunderstood by the inclusion of stimuli that require multiple cognitive processes (e.g., stimuli that require the processing of both facial and lexical information). For example, several studies of emotional control processes utilize an emotional face-word Stroop task where emotional words are overlaid on pictures of emotional faces and participants are asked to identify the affect expressed on the face [9,11-16]. Conflict is generated when the lexical properties of an emotional word (cognitive) conflicts with the affect depicted by the face (emotional). For example, a happy face overlaid with the word “fear”; this paradigm is also sometimes referred to as the emotional face-Stroop paradigm. Thus, an examination of conflicting emotional stimuli that does not include a lexical stimulus component may help clarify how and whether emotional conflict strategically adjusts processing priorities.

The emotional face-Stroop paradigm is useful and has led to many interesting and useful findings in emotional control [7]; however, multiple studies show dissociable systems in the processing of emotional faces and emotional words. For example, Rellecke et al. [17] used event-related potentials (ERPs) to show early processing of emotional stimuli, specifically the early posterior negativity (EPN), that was present when processing emotional facial stimuli, but not emotional word stimuli. They attributed their findings to the automatic processing of the more evolutionarily-prepared content in faces, relative to the more symbolic aspects of reading words. In two studies [18,19], Halgren et al. used depth electrodes in humans to show significantly different spatial and temporal properties of word and face processing, with face processing occurring earlier and across a larger variety of brain areas until later processing when multiple anatomical areas and functions converge. However, more recent fMRI work comparing face and word conflict showed faster processing of word-reading stimuli than face-related stimuli [20]. Finally, a large body of fMRI studies shows separable neuroanatomy for the processing of face versus word stimuli, with faces processed (among other areas) in the right lateral fusiform area and words processed in the left middle fusiform [21-23]. Based on these and other studies it is possible that emotional activation may be stimulus-dependent and differ for word or face stimuli.

Emotional face and emotional word stimuli are also associated with separable behaviors on Stroop-like tasks. For example, in one of the earlier studies of emotional face/word incongruencies, Raccuglia and Phaf [24] overlaid emotional word and emotional face stimuli to show increased response time interference when emotional words were used as distractors on face targets relative to when face distractors were used on word targets. There is a considerable literature on hemispheric asymmetries in lexical versus face emotion processing, with considerable evidence of left dominance for lexical processing and right dominance for facial processing [25]. Further evidence comes from a paradigm using an emotional face-word Stroop and a verbal-emotional Stroop. Presentation of emotional faces versus emotional words showed quantitatively different levels of interference in individuals induced to feel sad mood, with the verbal domain showing more attentional interference than face domain [26]. Incongruent emotional word distractors can also disproportionately bias recall of certain emotional faces, with increased recall for angry faces paired with emotional words (whether positive or negative) relative to angry faces paired with neutral or no words [27]. Finally, repetition effects that influence subsequent memory performance differ for face and word stimuli [28], with repetition seeming to influence memory for lexical stimuli more than face stimuli. This latter finding is of particular relevance as repetition priming is thought to be a strong contributor and possible alternative explanation for conflict adaptation effects [29].

Given the possible dissociable neural patterns between word and face stimuli, a novel examination of conflicting emotional processes that uses stimuli from within a similar domain (i.e., only conflicting face stimuli) may help clarify whether emotional control strategically adjusts processing priorities following emotional conflict. Thus, we designed an emotional conflict-laden task to examine if an emotional face task is sufficient to trigger conflict processing and subsequent adaptation (i.e., sequential-trial or Gratton effects) to incongruent and congruent emotions in the absence of lexical stimuli.

In order to examine emotional conflict processing and adaptation in the absence of a lexical component, we designed a novel paradigm in which the eyes and mouths of angry and happy faces were combined and mixed to investigate the effects of emotional conflict on the recruitment of control processes. We chose to use faces because of the evolutionary implications with one of the phylogenetically oldest form of social communication [30,31]. Previous findings indicate that eye whites alone are sufficient to elicit increased amygdala activation [32] and that processing the eye region conveys threat to the same extent as processing the entire face [33]. As such, angry eyes combined with a happy mouth (incongruent, high conflict) should activate emotional conflict mechanisms more than angry eyes combined with an angry mouth (congruent, low conflict). We used only the eyes and mouths to ensure that the task-relevant target (i.e., the eye whites) was prominent for each stimulus. We hypothesized that conflict elicited by emotional stimuli (eyes, mouth) would activate conflict evaluation processes and subsequent recruitment of emotional control. As a result of the implementation of control, and consistent with previous studies in cognitive conflict adaptation [4,5,8], incongruent trials followed by incongruent trials (iI trials) should be associated with decreased conflict activation relative to incongruent trials preceded by congruent trials (cI) trials. In order to examine the neural time course of conflict monitoring and adaptation processes ERPs were investigated in the current examination in addition to behavioral indices. A data reduction technique, temporospatial principal components analysis (PCA), was employed to identify the specific ERP factors sensitive to conflict monitoring and adaptation processes. Following the identification of the PCA factor sensitive to these processes, source localization was conducted to implicate a possible neural generator of the observed ERP activity.

## Materials and Methods

### Ethics Statement

All participants provided written informed consent as approved by the Brigham Young University Institutional Review Board in accord with all principles expressed in the Declaration of Helsinki.

### Participants

Participants were recruited from undergraduate psychology courses. Exclusion criteria, assessed via participant self report, included current or previous diagnosis of a psychiatric disorder, psychoactive medication use, substance use or dependence, neurological disorders, head injury, left-handedness, or uncorrected visual impairment. Final study enrollment included 41 individuals (29 female, 12 male) between the ages of 18 and 28 (*M* = 19.34, *SD* = 2.23).

### Computerized Task

**Figure 1:**
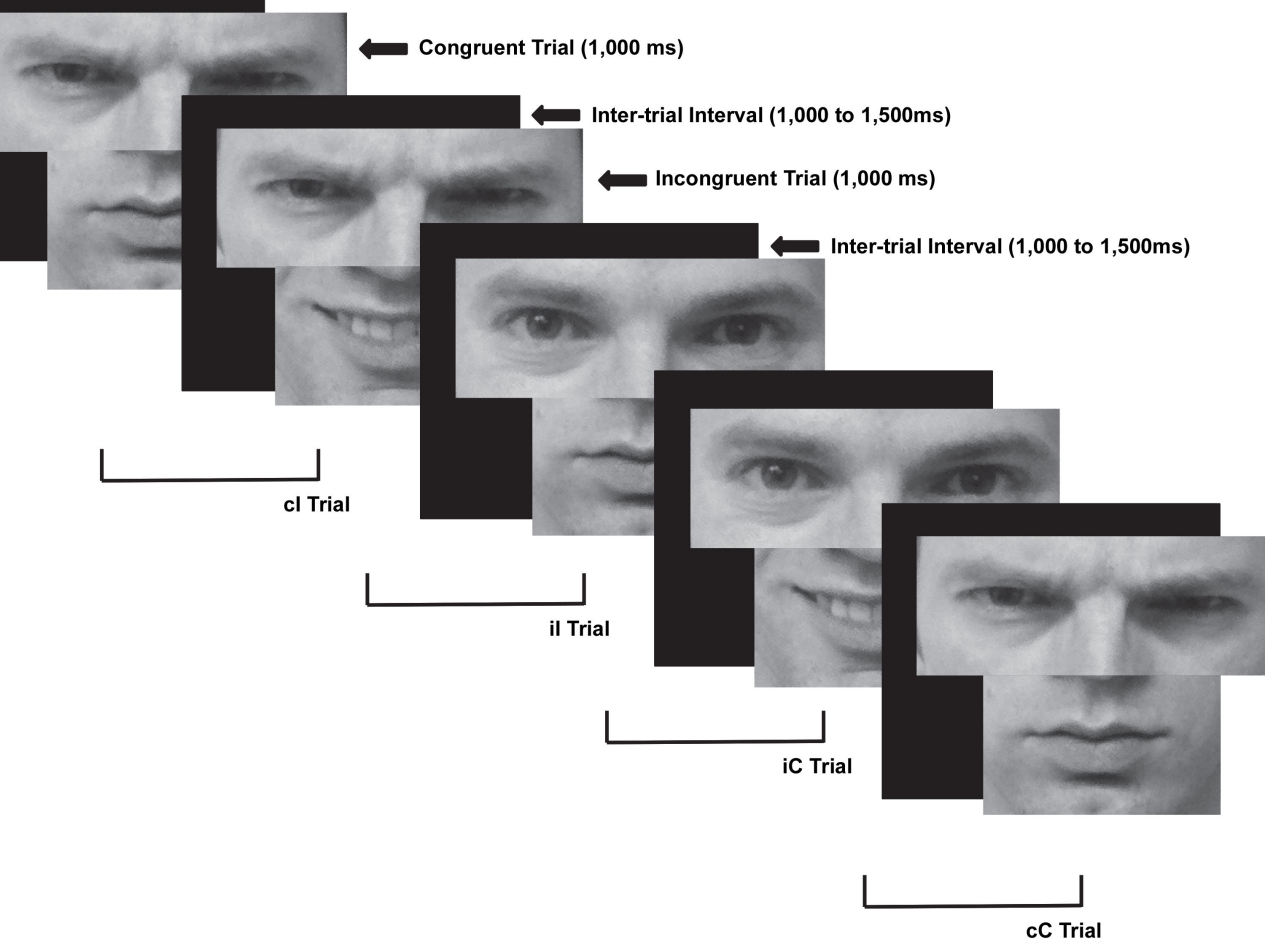
Face conflict paradigm example. Example of congruent and incongruent trials as well as trial combinations for conflict adaptation effects. The individual in the picture is not from the Ekman faces, but is an example. The individual has given written informed consent, as outlined in the PLOS consent form, to publication of their photograph. cC = congruent trial preceded by a congruent trial, cI = incongruent trial preceded by a congruent trial, iC = congruent trial preceded by an incongruent trial, iI = incongruent trial preceded by an incongruent trial.

Participants completed a novel emotional face-Stroop task (Figure 1). Each trial consisted of either angry or happy eyes and an angry or happy mouth that were congruent (eyes and mouth depicted the same emotion) or incongruent (eyes and mouth depicted different emotions). Three happy and angry male and three happy and angry female faces were chosen from the Ekman series faces [34] and were matched on brightness, contrast, and size (angry pictures: 38, 53, 69, 80, 96, 106; happy pictures: 34, 48, 66, 74, 93, 100). Angry and happy faces were chosen to provide an easy discrimination of stimuli that affect both the mouth and eyes and elicit strong emotional responses on either end of the approach versus avoid continuum [35]. At a viewing distance of approximately 20 inches, eye stimuli subtended 5°7’ horizontally and 2°32’ vertically; mouth stimuli subtended 5°7’ horizontally and 2°38’ vertically. Participants were shown the full happy and angry faces prior to beginning the task and asked to correctly identify the emotions presented to ensure participants understood the emotions depicted. During the experiment only the eyes and mouth were shown from the happy and angry faces; the eyes and mouth were always from the person shown in the image and never intermixed (e.g., eyes from one male were never combined with a mouth from a different male). Participants were instructed to respond as quickly and accurately as possible to the emotion portrayed by the eyes. An index-finger button press was used if the eyes depicted an angry emotion and a middle-finger button press was used if the eyes depicted a happy emotion (responses were counterbalanced across subjects). Stimuli remained on the screen for 1,000 ms. To decrease expectancy effects, the inter-trial interval varied randomly between 1,000 ms and 1,500 ms. Five blocks of 96 trials (480 total trials) were presented. Congruent and incongruent trials were equiprobably presented in each block.

To ensure that participants accurately understood the task stimuli, participants were shown the eyes and mouths of each face for both happy and angry images following task completion; participants were shown congruent trials only (i.e., the eyes and mouth always depicted the same emotion). Participants were asked to identify whether the eye-mouth combination represented a happy or angry emotion. Participants’ accuracy ranged from 75% to 100% with a mean of 89% and a standard deviation of 6%. A one-sample *t*-test against 50% was significant, *t*(40)=39.65, *p*<.0001, indicating that accuracy performance was above chance. These findings suggest that participants were able to accurately identify the emotion depicted by each face.

### Electroencephalogram Recording and Reduction

Electroencephalogram (EEG) was recorded from 128 scalp sites using a geodesic sensor net and Electrical Geodesics, Inc. (EGI; Eugene, OR) amplifier system (20K nominal gain, bandpass = .10-100 Hz). During recording, EEG was referenced to the vertex electrode and digitized continuously at 250 Hz with a 24-bit analogue-to-digital converter. Impedances were maintained below 50 kΩ. Data were digitally low-pass filtered at 30 Hz.

Correct-trial data were segmented as a function of previous- and current-trial congruencies (cC, cI, iC, iI). Individual-subject stimulus-locked averages were calculated using a window from -250 ms prior to stimulus presentation to 1,000 ms following stimulus presentation. Waveforms were baseline corrected using a 200 ms window from -250 ms to -50 ms prior to stimulus presentation. Eye blinks were removed from the segmented waveforms using independent components analysis (ICA) in the ERP PCA Toolkit [36]. The ICA components that correlated at .9 with the scalp topography of two blink templates were removed from the data [37]. Trials were considered bad if more than 15% of channels were marked bad. Channels were marked bad if the fast average amplitude exceeded 100 µV or if the differential average amplitude exceeded 50 µV. Data were average rereferenced and used the polar average reference effect (PARE) correction to correct for undersampling of the undersurface of the head [38].

To extract ERP components related to conflict monitoring and adaptation processes, temporospatial PCA was conducted using the ERP PCA Toolkit [36,39] with ERP data from cC, iC, iI, and cI trials. We followed previously published guidelines to extract ERP components [39-42]. A temporal PCA with promax rotation using all time points from single subject averages as variables with participants, trials, and all electrodes as observations was first conducted and yielded 10 temporal factors (TFs). A spatial PCA with infomax rotation using all electrode sites as variables and participants, trials, and temporal factors as observations followed and yielded 5 spatial factors (SFs) [40,42].

**Figure 2:**
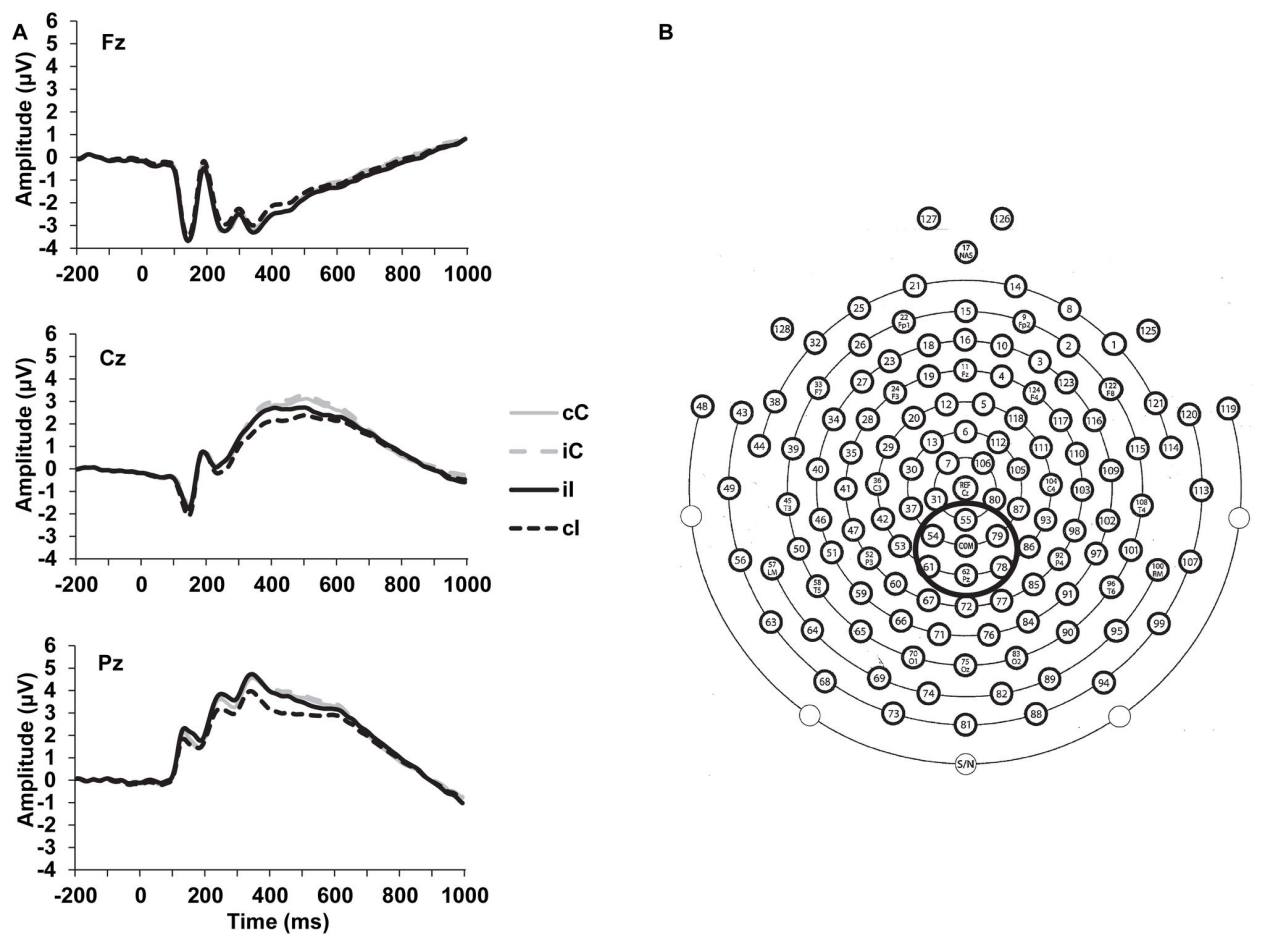
Event-related potential waveforms for midline electrodes and sensor layout. a) Grand average waveforms depicting ERP activity at Fz (top), Cz (middle), and Pz (bottom). b) Sensor layout of the 128-channel geodesic sensor net. Solid-line circle indicates centro-parietal recording sites averaged for the early and late P3-like components. cC = congruent trial preceded by a congruent trial, cI = incongruent trial preceded by a congruent trial, iC = congruent trial preceded by an incongruent trial, iI = incongruent trial preceded by an incongruent trial.

In order to avoid assumptions about the present ERP data, a bottom-up, data-driven approach was implemented to identify factors for subsequent analysis. Factors were chosen based on whether the factor’s variance accounted for at least 2.5% of the variance in the overall waveform. The purposes of this procedure were three-fold. First, this approached served to screen out factors that accounted for minimal amount of variance in the overall waveform (i.e., EEG noise) and retain only those factors that influenced the cC, iC, iI, and cI ERP waveforms. Second, considering the exploratory nature of this study, this approach minimized the number of statistical comparisons performed to decrease the chance of committing Type I errors. Third, this approach yielded only the potential factors that would represent meaningful TFSFs for the current examination of whether ERPs are sensitive to the conflict monitoring and adaptation processes. This automated, data-driven approach yielded nine TFSFs that accounted for at least 2.5% of the variance in the ERP waveform. The nine TFSFs with the corresponding peak channel and latency are presented in Table 1 (for sensor layout see Figure 2). The instantaneous amplitude at the peak amplitude was extracted for subsequent analysis.

**Table 1:** Mean response time (ms), error rate, and amplitude (μV) summary data.

Temporospatial Factor (TFSF)	Peak Channel	Peak Latency (ms)
TF1SF1	75 (Oz)	406
TF1SF2	8	406
TF1SF3	55 (CPz)	406
TF2SF1	81	854
TF2SF2	32	854
TF2SF3	126	854
TF3SF1	90	242
TF4SF1	25	574
TF5SF1	70	146

After identifying the temporal and spatial characteristics of potential ERP components that showed conflict adaptation effects, single subject averages were examined. A temporospatial PCA was first employed before analyzing the single subject averages to identify the temporal and spatial characteristics of ERP activity that was associated with conflict adaptation effects. This bottom-up approach minimizes bias that may arise from choosing ERP activity for analysis based solely on visual inspection of observed ERP waveforms. A mean amplitude approach was used to quantify ERP components to avoid the potential biasing effects of background EEG noise [43]. Mean amplitude was extracted from 250 to 400 ms and averaged across six centro-parietal electrodes (54, 55, 61, 62 [Pz], 78, 79; see Figure 2) to quantify the early P3-like component. Mean amplitude activity was also extracted across the same centro-parietal electrodes from 400 to 600ms to quantify the late P3-like component.

### Data Analysis

To reduce the possibility of Type I error [44], robust analyses of variance (ANOVAs) were conducted using the ERP PCA Toolkit [36,45]. Robust statistics are more conservative than conventional ANOVAs and help avoid erroneous findings from inflated Type I error rates. To decompose significant interactions, Fisher’s least significant difference approach was used to control for family-wise Type I error when decomposing significant interactions. Considering the exploratory nature of the study and conservative nature of robust ANOVAs, *p*-values below .05 were considered significant. The seed for the number generation was set at 1,000, and the number of iterations used for bootstrapping was 50,000 [37,46].

A competing theoretical explanation of conflict adaptation effects indicates that improved behavioral performance following conflict may primarily be result of stimulus-response repetitions [29]. Repetitions of exact face-mouth combinations were improbable. Indeed, an analysis of the data collected after study completion indicated that an average of 3.09% (standard deviation = 0.76%) of trials presented contained an exact trial repetition. Thus, it is unlikely that stimulus-response repetition effects biased the current findings; however, behavioral data assessed whether conflict adaptation was biased by the inclusion of response repetitions by examining data including and omitting response repetitions. Considering that previous research indicates that conflict adaptation effects remain in ERP data after the exclusion of response repetitions [47,48], to maximize the signal-to-noise ratio for ERP data averaging and for subsequent source analysis trials with response repetitions were included in ERP analyses.

Response time (RT) data were calculated excluding omitted-response trials. For RTs and ERPs, error trials and post-error trials were also excluded due to previous research showing faster RTs on error trials and slower RTs on post-error trials [47,49]. Separate robust Repetition (data including response repetitions, data omitting response repetitions) x Previous-trial Congruency (congruent, incongruent) x Current-trial Congruency (congruent, incongruent) ANOVAs were conducted for RTs and error rates. Previous-trial Congruency x Current-trial Congruency robust ANOVAs were conducted on the conflict adaptation temporospatial factors and single subject average ERP data. A congruency effect was defined as a significant difference between congruent and incongruent trials for the current trial only (i.e., main effect of current-trial congruency). Conflict adaptation effects were considered significant when the Previous-trial Congruency x Current-trial Congruency interaction was significant and was confirmed with a significant cI/iI trial difference [48].

Source analysis was conducted using a four-shell model in FieldTrip, an open-source toolbox for Matlab [50]. Considering that source localization was conducted on factor data, the entire epoch was utilized for localization. A position of maximum fit used a maximum-likelihood estimation algorithm [51]. Solution stability was assessed using a jackknife technique that recomputed the second spatial PCA solution 41 times, each time excluding one participant, and conducting a source analysis on the solution.

## Results

### Behavioral

Mean RT and error rate data are presented in Table 2. The Repetition x Previous-trial Congruency x Current-trial Congruency robust ANOVA indicated that RTs showed a congruency effect and were longer for incongruent trials relative to congruent trials, *T*_WJt_/c(1.0,40.0)=115.44, *p*<.00000001. The Previous-trial Congruency x Current-trial Congruency interaction was significant, *T*_WJt_/c(1.0,40.0)=9.71, *p*=.0041. RTs were shorter for congruent trials preceded by congruent trials (cC) than for congruent trials preceded by incongruent trials (iC), *T*_WJt_/c(1.0,40.0)=15.04, *p*=.0004; no differences were shown for cI and iI trials suggesting that significant conflict adaptation effects were not observed for RTs, *T*_WJt_/c(1.0,40.0)=0.08, *p*=.77. The Repetition x Previous-trial Congruency x Current-trial Congruency interaction was not significant, *T*_WJt_/c(1.0,40.0)=2.73, *p*=.11, indicating that conflict adaptation effects were not significantly influenced by repetition priming.

**Table 2:** Mean response time (ms) and error rate summary data for trials omitting and including response repetitions.

	Omitting Response Repetitions		Including Response Repetitions	
	Mean	SD	Mean	SD
cC RT	667	50	662	49
iC RT	678	54	673	58
iI RT	705	52	693	54
cI RT	699	52	701	51
cC error rates	22%	12%	22%	9%
iC error rates	22%	10%	24%	10%
iI error rates	35%	16%	38%	13%
cI error rates	36%	13%	41%	13%

Note. RT = response time; cC = congruent trial preceded by a congruent trial; iC = congruent trial preceded by an incongruent trial; iI = an incongruent trial preceded by an incongruent trial; cI = incongruent trial preceded by a congruent trial.

A robust ANOVA on error rates indicated that error rates were higher for incongruent trials relative to congruent trials, *T*_WJt_/c(1.0,40.0)=57.01, *p*<.00000001. The Previous-trial Congruency x Current-trial Congruency interaction was significant, *T*_WJt_/c(1.0,40.0)=10.67, *p*=.012. Error rates were larger for cI trials compared to iI trials confirming that error rate data showed significant conflict adaptation effects, *T*_WJt_/c(1.0,40.0)=8.36, *p*=.044; no differences were shown for cC and iC trials, *T*_WJt_/c(1.0,40.0)=1.50, *p*=.23. The Repetition x Previous-trial Congruency x Current-trial Congruency interaction for error rates was not significant, *T*_WJt_/c(1.0,40.0)=1.19, *p*=.28.

### Event-Related Potentials

**Figure 3:**
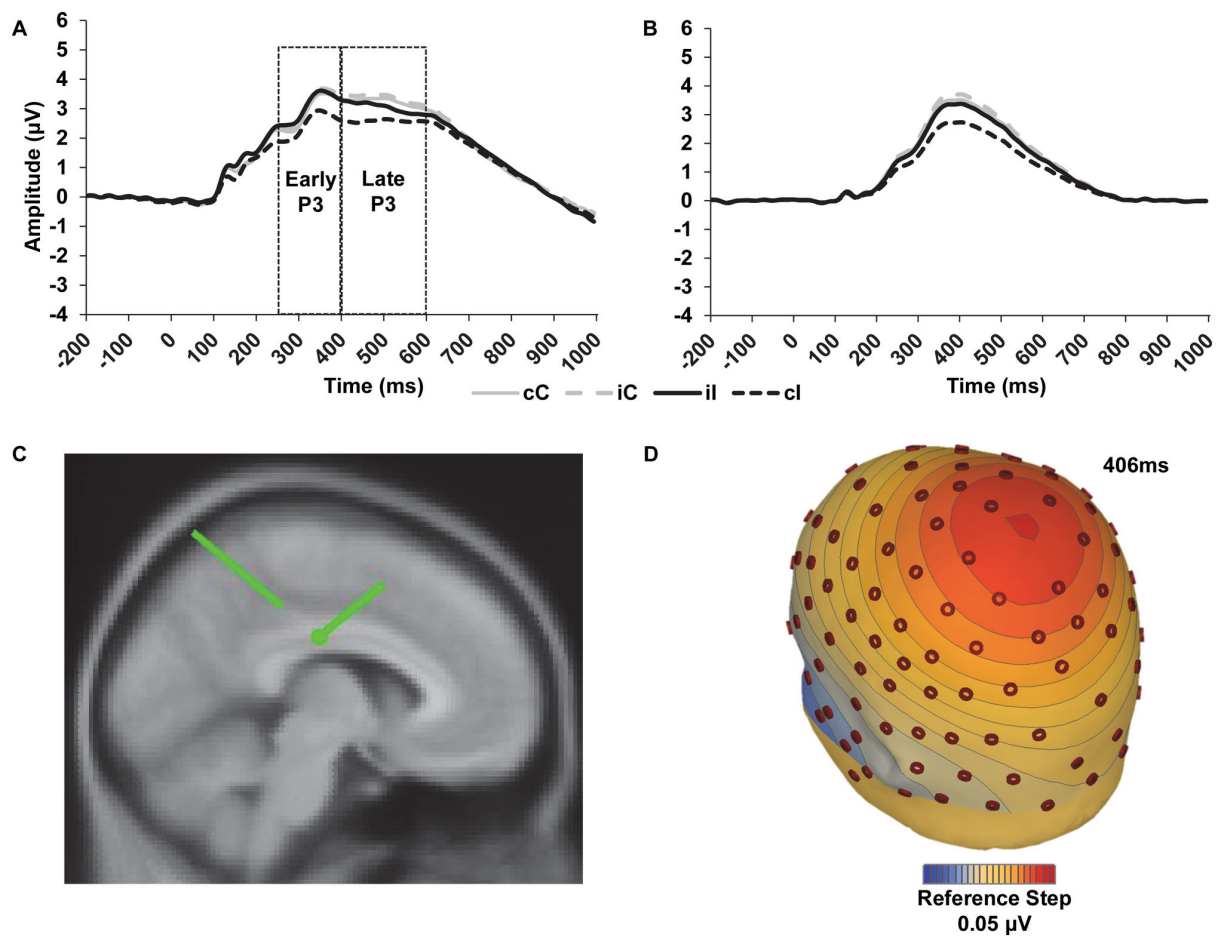
Event-related potential waveforms and source localization. a) Grand average waveforms depicting P3 activity average across six centro-parietal recording sites as a function of previous- and current-trial congruency. Dotted lines indicate time windows used to extract the early and late P3 activity. b) Portion of P3 captured by temporal factor 1 spatial factor 3 (TF1SF3) as a function of previous- and current-trial congruency and extracted from activity at CPz. c) Sagittal view of dipole source for TF1SF3. d) Voltage map for TF1SF3 iI minus cI activity. cC = congruent trial preceded by a congruent trial, cI = incongruent trial preceded by a congruent trial, iC = congruent trial preceded by an incongruent trial, iI = incongruent trial preceded by an incongruent trial.

We first present the data for the temporospatial PCA factors. After artifact correction and rejection, correct-trial ERPs contained an average ± standard deviation (minimum to maximum) of 113 ± 34 (42 to 170) cC trials, 51 ± 17 (16 to 80) iC trials, 51 ± 16 (22 to 82) cI trials, and 42 ± 17 (12 to 76) iI trials. The Previous-trial Congruency x Current-trial Congruency robust ANOVA on TF1SF3 amplitude yielded a main effect of current-trial congruency with more negative amplitude for incongruent compared to congruent trials, *T*_WJt_/c(1.0,40.0)=16.26, *p*=.0003. Notably, the Previous-trial Congruency x Current-trial Congruency interaction was significant suggesting that TF1SF3 may show significant conflict adaptation effects, *T*_WJt_/c(1.0,40.0)=7.55, *p*=.0093 (see Figure 3). Less positive amplitudes were shown for cI trials compared to iI trials confirming that TF1SF3 showed significant conflict adaptation effects, *T*_WJt_/c(1.0,40.0)=17.51, *p*=.0002; no differences were shown for cC and iC trials, *T*_WJt_/c(1.0,40.0)=3.78, *p*=.06. Mean ERP TF1SF3 amplitude data are presented in Table 3.

**Table 3:** Amplitude (μV) summary data.

	Mean	SD
cC Early P3 amplitude	2.7	1.9
iC Early P3 amplitude	2.8	1.9
iI Early P3 amplitude	2.8	2.0
cI Early P3 amplitude	2.4	1.8
cC Late P3 amplitude	3.2	1.7
iC Late P3 amplitude	3.4	1.7
iI Late P3 amplitude	3.0	1.8
cI Late P3 amplitude	2.7	1.6
cC TF1SF3 amplitude	3.5	1.9
iC TF1SF3 amplitude	3.7	1.9
iI TF1SF3 amplitude	3.3	1.9
cI TF1SF3 amplitude	2.7	1.7

Note. cC = congruent trial preceded by a congruent trial; iC = congruent trial preceded by an incongruent trial; iI = an incongruent trial preceded by an incongruent trial; cI = incongruent trial preceded by a congruent trial

None of the remaining eight TFSFs yielded a significant main effect of current-trial congruency (Ts<3.4, *p*s>.08) or a significant Previous-trial Congruency x Current-trial Congruency interaction (Ts<2.9, *p*s>.09). Taken together, only one of the nine TFSFs was sensitive to conflict monitoring and adaptation processes, showing a significant congruency effect and significant conflict adaptation effects. TF1SF3 was then chosen for subsequent analysis and source localization. Considering the P3-like characteristics of TF1SF3, it will be subsequently referred to as the P3 factor.

For the more traditional ERP measurements, mean ERP amplitudes are shown in Table 3. The grand average waveform for the early and late P3 is shown in Figure 3 (additional ERP grand average waveforms for Fz, Cz, and Pz electrodes are shown in Figure 2 with the sensor layout). The follow-up Previous-trial Congruency x Current-trial Congruency analysis on the early P3-like component from the single subject averages yielded a nonsignificant main effect of current-trial congruency, *T*_WJt_/c(1.0,40.0)=1.49, *p*=.02. The Previous-trial Congruency x Current-trial Congruency interaction was also not significant, *T*_WJt_/c(1.0,40.0)=3.18, *p*=.08. A main effect was observed for the Previous-trial Congruency x Current-trial Congruency analysis on the late P3-like component indicating a significant congruency effect, *T*_WJt_/c(1.0,40.0)=14.30, *p*=.0009. Amplitude was more positive for congruent trials compared to incongruent trials. The Previous-trial Congruency x Current-trial Congruency interaction was not significant, *T*_WJt_/c(1.0,40.0)=1.08, *p*=.30.

Finally, we conducted source localization on the current data. As evident in the grand average waveform based on visual inspection as well as the P3 factor, incongruent trials were less positive than congruent trials; cI trials were also less positive than iI trials for the P3 factor (see Figure 3). Source localization was conducted on the iI minus cI difference wave to implicate a possible neural generator involved in the affective conflict resolution of the P3 factor. The medial cingulate gyrus was identified as the likely neural generator (two symmetrical dipoles with Talairach coordinates of 11.9, -18.5, 29.8 and -11.9, -18.5, 29.8—see Figure 3). Residual variance of the dipoles was 3.88%. Jack-knife analysis was consistent with overall findings and indicated that dipoles were clustered in the cingulate gyrus for 40 of the 41 jack-knife solutions. The remaining jack-knife solution was localized to the inferior parietal lobe (coordinates = 42.2, -22.2, 26.8). The jack knife analysis further revealed that the left-hemisphere cingulate dipole was larger than the right-hemisphere cingulate dipole, *t*(26)=-6.80, *p*<.0001.

## Discussion

Previous paradigms employed for the investigation of affective control processes relied on a combination of symbolic emotional word stimuli and affectively salient facial expressions [9,11-15]. The current examination used a novel paradigm with only congruent or incongruent facial expressions that was sensitive to emotional conflict evaluation and resolution for both behavioral (i.e., error rate) and ERP data. These findings are important for three reasons. First, the presence of an incongruency-related interference (i.e., congruency effect) with four dependent variables of interest (RTs, error rates, ERP TF1SF3 [P3 factor], late P3-like component) indicates conflict is present when individuals see discordant facial expressions. Second, the presence of emotional conflict adaptation in the absence of word stimuli for error rates and the P3 factor indicate that the lexical and symbolic properties of the words were not the only driving force behind emotional conflict adaptation seen in previous studies using the face-Stroop paradigm [6,11,52]. Third, current findings support the notion that emotionally-conflicting stimuli can trigger behaviors in a pattern similar to cognitive conflict [5], supporting the notion that emotional control and cognitive control are drawing from a similar pool of cognitive resources [10].

Event-related potentials were used to examine the spatiotemporal pattern of neural activity associated with emotional conflict. Current findings showed an ERP TFSF component that was similar to a P3 in latency and amplitude, with peak activation around 410ms following stimulus presentation and that was more positive for congruent trials, when eyes and mouth depicted the same emotion, than for incongruent trials, when eyes and mouth depicted conflicting emotions. Furthermore, the P3 factor exhibited conflict adaptation effects such that cI trials were less positive than iI trials indicating it reflects processes sensitive to the top-down recruitment of control following emotional conflict. The examination of the P3 factor represented an underlying component of the early and late P3. Other examinations of the P300 using PCA have been effective separating out the Novelty P3 and Slow Wave components underlying the P300 ERP [53,54] as well as the P200, P300, Slow Wave, and reward-related components associated with the feedback negativity [55], demonstrating the utility of the temporospatial PCA in analyzing the component structure of ERPs. Although the early and late P3 examined in the current investigation were not sensitive to conflict adaptation effects and only the late P3 showed a significant congruency effect, a strength of the current study was that the temporospatial PCA was able to isolate a P3 factor from overlapping ERP activity that was modulated by conflict adaptation effects. Furthermore to the extent to which overlapping ERP component activity was isolated from the P3 factor, the medial cingulate gyrus was implicated in the generation of the P3 factor. In short, the P3 factor provides a likely candidate for the examination of affective control processes during an emotional conflict paradigm.

In cognitive conflict adaptation studies using a traditional Stroop color-word task, ERP investigations primarily focused on the conflict slow potential (SP) and the Stroop N450 [56,57]. The conflict SP is more positive for incongruent than for congruent trials and for cI compared to iI trials. Similar to the P3 factor in our study, however, the N450 is less positive for incongruent trials relative to congruent trials. Source localization studies implicated the ACC as the neural generator of the N450 [58]. The N450 may be a delayed occurrence of the conflict N200 and shows a similar scalp topography and component latency to the P3 factor in the current study [58,59]. The conflict N200 shows a similar scalp topography to the N450 and our P3 factor and is less negative for congruent trials than for incongruent trials and for iI compared to cI trials [47,48]; the ACC is also implicated in the generation of the N2 [60]. In contrast to the conflict N200, the P3 factor show reversed polarity—incongruent trials were more negative than congruent trials and showed more posterior source localization. In sum, however, it seems that the P3 factor to emotional face stimuli in this instance fits in well with the conflict-monitoring family of ERP components, such as the Stroop N450 and conflict N200.

The cingulate gyrus was implicated in the generation of the P3 factor in the current study, which is generally consistent with conflict monitoring accounts, but the current P3 factor was more posterior than what is typically seen in these studies [5,61]. Limitations of EEG source localization notwithstanding, other source localization studies of the NoGo P3 elicited using tasks with emotionally-salient stimuli implicated the ACC in the neural generation of the NoGo P3 suggesting that the ACC is involved in emotional response inhibition [62-64]. The cingulate gyrus is also thought to be involved in emotional conflict evaluation and resolution in the model recently proposed by Etkin et al. [7], wherein greater pregenual ACC activation is sensitive to the recruitment of control and is associated with reductions in conflict activation of the amygdala [6]. Thus, emotional conflict may be suppressed by the cingulate through biasing control to focus processing on task-relevant stimuli and suppressing attention to task-irrelevant stimuli. Similar to models of cognitive control [5,61], the cingulate is involved in emotional conflict evaluation and signals the PFC for the recruitment of control to inhibit emotional conflict activation. In the emotional control network, however, the amygdala is also implicated in the evaluation of emotional conflict and is inhibited by the top-down implementation of control by the cingulate [9]. Although amygdala activation was not directly measured in the current study, these findings provide a novel paradigm for future fMRI studies to examine the emotional control system.

Present findings have implications for examining affective control in psychiatric disorders. For example, the cingulate is implicated in affective-regulation abnormalities in mood disorders [65] and psychotic disorders [66]. With regards to major depressive disorder (MDD) and generalized anxiety disorder (GAD), the current paradigm provides a framework in which emotional processing may be investigated in the absence of lexical stimuli, a limitation to previous research on emotional regulation in MDD and GAD [11,12]. In addition to cingulate dysfunction, amygdala abnormalities have also been implicated in depression [65] and anxiety [52] suggesting that it may be possible that a “double jeopardy” effect of cingulate and amygdala dysfunction interfere with the system’s modulation emotional control. Studies examining affective and cognitive control using traditional conflict-related paradigms (e.g., Flanker or Stroop color-word tasks), the present paradigm, as well as the widely used emotional face-word Stroop task, would elucidate possible cognitive and affective dysfunction separately in MDD and GAD while complimenting the previously demonstrated abnormalities at the cognition-emotion control interface [11,12]. Considering that cognitive and emotional control compete for similar attentional resources [10], it further remains unclear as to whether previously shown impairments in control regulation in MDD and GAD are related to emotional or cognitive control processing deficits [67].

In light of the burgeoning interest for examining reciprocal connections and interactions of cognitive and affective control, the present paradigm provides a promising task for investigating the trial-by-trial recruitment of affective control in the absence of cognitively demanding stimuli. Although participants made a cognitive classification as to the affective valence of the eyes in the paradigm, lexical or semantic stimuli were not utilized to generate stimuli conflict. In regards to previous studies of affective control that used tasks with both lexical and emotional stimuli, it remains unclear whether the implicated brain regions are unique to affective control or are specifically related to the interaction of lexical and affective control processes. A more solid affective-control groundwork is requisite to further understanding of neural processes specific to the interaction of cognitive and affective control. Indeed, a greater understanding of affective control is needed to begin clarifying how emotional processes impair or enhance cognitive control function.

The present study also has a number of potential limitations. Participants were shown each angry and happy face prior to beginning the task to ensure understanding of the emotion depicted by each face then subsequently identified whether each face was angry or happy following the task based on just the eye-mouth combination; however, participants did not independently identify the eye whites for each emotion and face. Although eye whites alone have been shown to be sufficient to elicit increased amygdala activation [32] and that processing the eye region alone conveys threat to the same extent as processing the entire face [33], future examinations should ensure that participants can accurately identify the emotional valence depicted based on the eye region alone. Furthermore, the complete neural network involved in the recruitment of attentional processes in response to emotional conflict could not be examined in the current investigation; however, the present study provides a potential paradigmatic framework for investigating the emotional control network without lexical stimuli. Future research will hopefully examine the similarities and differences in the emotional and cognitive control networks in isolation to provide a firm background for the investigation of the interaction of the cognitive and emotional control networks.

In sum, the current examination showed significant conflict interference and conflict adaptation using a face-only paradigm with no word stimuli. There was a P3 factor that was more positive for congruent trials than for incongruent trials and was sensitive to the top-down recruitment of affective control following emotional conflict. The cingulate gyrus was implicated in the generation of the P3 factor which is consistent with current understanding of the emotional control network [7]. Further, this study novel paradigm in which emotional control may be investigated without the potential for confounding symbolic word stimuli and affective face stimuli. 
